# 3-Amino-4-(1,3-benzoxazol-2-yl)-5-(cyclo­hexyl­amino)­thio­phene-2-carbo­nitrile

**DOI:** 10.1107/S1600536813019880

**Published:** 2013-07-27

**Authors:** Chiraz Youssef, Mohamed Belhouchet, Hamed Ben Ammar, Ridha Ben Salem

**Affiliations:** a Laboratoire de Chimie Organique Physique, Département de Chimie, Faculté des Sciences de Sfax, Université de Sfax, BP 1171, 3000 Sfax, Tunisia; b Laboratoire Physico-Chimie de l’Etat Solide, Département de Chimie, Faculté des Sciences de Sfax, Université de Sfax, BP 1171, 3000 Sfax, Tunisia

## Abstract

In the title compound, C_18_H_18_N_4_OS, the cyclo­hexyl ring adopts a chair conformation. The other rings of this compound lie almost in the same plane, with a mean deviation of 0.03 (2) Å from the least-squares plane defined by the 14 constituent atoms. There are intra­molecular N—H⋯N and N—H⋯O hydrogen bonds, as well as inter­molecular N—H⋯N hydrogen bonds, which link the mol­ecules into centrosymmetric dimers.

## Related literature

For the pharmacological and biological activities of benzoxazole derivatives, see: Isomura *et al.* (1983 ▶); Cheng *et al.*(1993 ▶); Koci *et al.* (2002 ▶); Hoffman *et al.* (1993 ▶); Arpaci *et al.* (2002 ▶). For the synthesis and a similar structure, see: Youssef *et al.* (2011 ▶); Belhouchet *et al.* (2012 ▶).
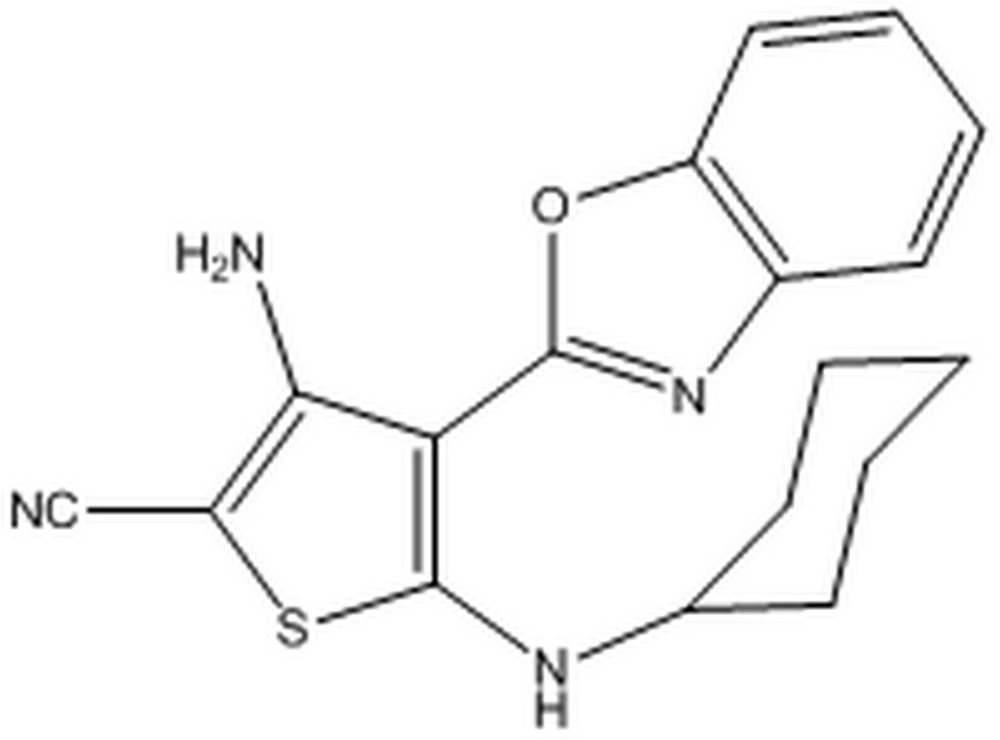


## Experimental

### 

#### Crystal data

C_18_H_18_N_4_OS
*M* *_r_* = 338.42
Monoclinic, 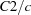

*a* = 24.270 (5) Å
*b* = 6.193 (5) Å
*c* = 23.578 (5) Å
β = 107.554 (5)°
*V* = 3379 (3) Å^3^
*Z* = 8
Mo *K*α radiation
μ = 0.20 mm^−1^
*T* = 293 K
0.3 × 0.25 × 0.22 mm

#### Data collection

Bruker APEXII area-detector diffractometer
Absorption correction: multi-scan (*SADABS*; Sheldrick, 1996 ▶) *T*_min_ = 0.655, *T*_max_ = 0.746
13506 measured reflections
2961 independent reflections
1904 reflections with *I* > 2σ(*I*)
*R*_int_ = 0.049

#### Refinement

*R*[*F* ^2^ > 2σ(*F* ^2^)] = 0.042
*wR*(*F* ^2^) = 0.112
*S* = 1.01
2961 reflections
230 parameters
H atoms treated by a mixture of independent and constrained refinement
Δρ_max_ = 0.17 e Å^−3^
Δρ_min_ = −0.17 e Å^−3^

### 

Data collection: *APEX2* (Bruker, 2009 ▶); cell refinement: *SAINT* (Bruker, 2009 ▶); data reduction: *SAINT*; program(s) used to solve structure: *SHELXS97* (Sheldrick, 2008 ▶); program(s) used to refine structure: *SHELXL97* (Sheldrick, 2008 ▶); molecular graphics: *ORTEP-3 for Windows* (Farrugia, 2012 ▶) and *DIAMOND* (Brandenburg, 1998 ▶); software used to prepare material for publication: *publCIF* (Westrip, 2010 ▶).

## Supplementary Material

Crystal structure: contains datablock(s) I, global. DOI: 10.1107/S1600536813019880/bv2222sup1.cif

Structure factors: contains datablock(s) I. DOI: 10.1107/S1600536813019880/bv2222Isup2.hkl

Supplementary material file. DOI: 10.1107/S1600536813019880/bv2222Isup4.cml

Additional supplementary materials:  crystallographic information; 3D view; checkCIF report

## Figures and Tables

**Table 1:** Hydrogen-bond geometry (Å, °)

*D*—H⋯*A*	*D*—H	H⋯*A*	*D*⋯*A*	*D*—H⋯*A*
N2—H1*N*2⋯O1	0.92 (3)	2.13 (3)	2.783 (4)	127 (2)
N2—H2*N*2⋯N1^i^	0.86 (3)	2.25 (3)	3.088 (4)	166 (3)
N3—H1*N*3⋯N4	0.86 (2)	2.12 (3)	2.787 (3)	135 (2)

Symmetry code: (i) 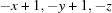.

**Fig. 1.**
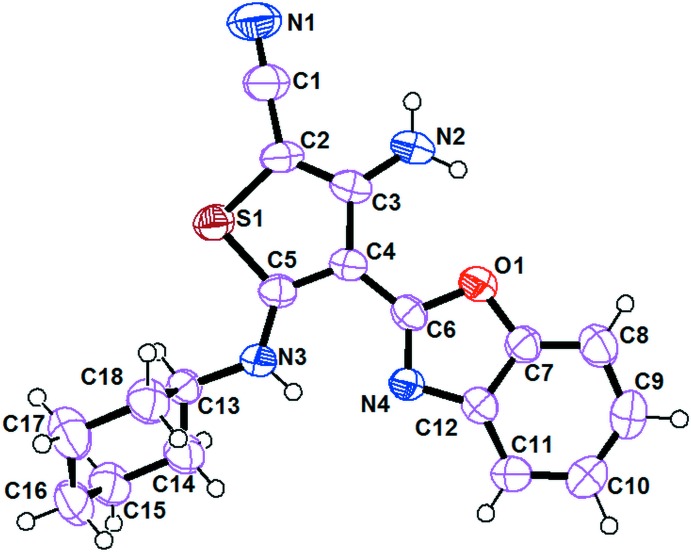
The molecular structure of the title compound, with displacement ellipsoids drawn at the 50% probability level. H atoms are shown as small spheres of arbitrary radius.

**Fig. 2.**
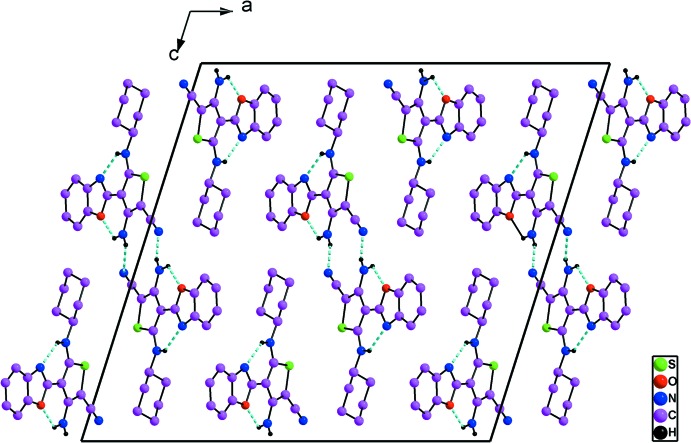
The crystal packing of the title compound, viewed down the b axis. Dashed lines indicate hydrogen bonds. H atoms not involved in hydrogen bonding have been omitted for clarity.

## supplementary crystallographic information

### Comment

Substituted thiophenes have attracted considerable interest because they are endowed with variety of biological activities and have wide range of therapeutic properties. The Literature survey indicates that benzoxazole derivatives possess different pharmacological and biological activities (Isomura *et al.*, 1983) which of most potent activity such as anti-tumor (Cheng *et al.* 1993), anticancer (Koci *et al.*, 2002), antiviral (Hoffman *et al.*, 1993), or antimicrobial (Arpaci *et al.*, 2002) properties. For these reasons we thought to synthesize thiophene system incorporating benzoxazole.

The present report describes the molecular structure of 3-amino-4-benzo[*d*]oxazol-2-yl-5-(cyclohexylamino) thiophène-2-carbonitrile. The crystal structure of this compound is built up from two fused five(O1/C6/N4/C12/C7) and six membered (C7—C11) rings linked to five (C2—C5/S1) membered ring *via* C6—C4 bond. The built planar entity (with a mean deviation of 0.03 (2) Å from the least square plane defined by the fourteen constituted atoms) is linked to the cyclohexyl ring (adopting a chair conformation) *via* two C—N bonds (C5—N3 and C13-N3) as shown in Figure 1. All bond lengths are normal and are comparable with those reported for a similar structure (Belhouchet *et al.*, 2012). There are intramolecular N—H···N and N—H···O hydrogen bonds as well as intermolecular N—H···N hydrogen bonds which link the molecules into centrosymmetric dimers (Figure 2 and Table 1). There are no other intermolecular interactions present.

### Experimental

Previously, we investigated the reaction of benzoxazole with active methylene compounds and aldehyde in alkaline medium, which has proved to be a convenient route for the synthesis of pyridobenzoxazole ring systems (Youssef *et al.*,2011). Now, we have extended our synthetic program to the synthesis of thiophenes ring system, utilizing benzoxazol-2-cyanomethyle as a key starting material.

To a cold suspension of potassium tertio-butylate (25 mmol) in THF (30 ml) benzoxazol-2-cyanomethyl was added (22 mmol) and followed by cyclohexyl isothiocyanate (22 mmol). The mixture was stirred overnight at room temperature and treated with the chloroacetone (22 mmol), stirring was continued for 4 h. The reaction mixture was poured onto ice cold water. Acidification using dilute HCl until the medium becomes acidic gave the synthesized solid product which was filtered off, washed with water, dried and recrystallized from aqueous ethanol solution to give single-crystal suitable for the X-ray diffraction.)

### Refinement

H atoms bonded to N2 and N3 were located in a difference Fourier map and refined freely. Other H atoms were positioned geometrically and allowed to ride on their parent atoms, with C—H = 0.93–0.98 Å, and with *U*_iso_(H) = 1.2*U*_eq_(C).)

### Crystal data

**Table:**

C_18_H_18_N_4_OS	*F*(000) = 1424
*M**_r_* = 338.42	*D*_x_ = 1.331 Mg m^−^^3^
Monoclinic, *C*2/*c*	Mo *K*α radiation, λ = 0.71073 Å
Hall symbol: -C 2yc	Cell parameters from 2961 reflections
*a* = 24.270 (5) Å	θ = 1.8–24.9°
*b* = 6.193 (5) Å	µ = 0.20 mm^−^^1^
*c* = 23.578 (5) Å	*T* = 293 K
β = 107.554 (5)°	Parallelepipedic, violet
*V* = 3379 (3) Å^3^	0.3 × 0.25 × 0.22 mm
*Z* = 8	

### Data collection

**Table:**

Bruker APEXII area-detector diffractometer	2961 independent reflections
Radiation source: fine-focus sealed tube	1904 reflections with *I* > 2σ(*I*)
Graphite monochromator	*R*_int_ = 0.049
φ and ω scans	θ_max_ = 24.9°, θ_min_ = 1.8°
Absorption correction: multi-scan (*SADABS*; Sheldrick, 1996)	*h* = −28→27
*T*_min_ = 0.655, *T*_max_ = 0.746	*k* = −7→7
13506 measured reflections	*l* = −27→27

### Refinement

**Table:**

Refinement on *F*^2^	Secondary atom site location: difference Fourier map
Least-squares matrix: full	Hydrogen site location: inferred from neighbouring sites
*R*[*F*^2^ > 2σ(*F*^2^)] = 0.042	H atoms treated by a mixture of independent and constrained refinement
*wR*(*F*^2^) = 0.112	*w* = 1/[σ^2^(*F*_o_^2^) + (0.0502*P*)^2^ + 0.4702*P*] where *P* = (*F*_o_^2^ + 2*F*_c_^2^)/3
*S* = 1.01	(Δ/σ)_max_ < 0.001
2961 reflections	Δρ_max_ = 0.17 e Å^−^^3^
230 parameters	Δρ_min_ = −0.17 e Å^−^^3^
0 restraints	Extinction correction: *SHELXL97* (Sheldrick, 2008), Fc^*^=kFc[1+0.001xFc^2^λ^3^/sin(2θ)]^-1/4^
Primary atom site location: structure-invariant direct methods	Extinction coefficient: 0.0009 (2)

### Special details

**Table:**

Geometry. All e.s.d.'s (except the e.s.d. in the dihedral angle between two l.s. planes) are estimated using the full covariance matrix. The cell e.s.d.'s are taken into account individually in the estimation of e.s.d.'s in distances, angles and torsion angles; correlations between e.s.d.'s in cell parameters are only used when they are defined by crystal symmetry. An approximate (isotropic) treatment of cell e.s.d.'s is used for estimating e.s.d.'s involving l.s. planes.
Refinement. Refinement of *F*^2^ against ALL reflections. The weighted *R*-factor *wR* and goodness of fit *S* are based on *F*^2^, conventional *R*-factors *R* are based on *F*, with *F* set to zero for negative *F*^2^. The threshold expression of *F*^2^ > σ(*F*^2^) is used only for calculating *R*-factors(gt) *etc*. and is not relevant to the choice of reflections for refinement. *R*-factors based on *F*^2^ are statistically about twice as large as those based on *F*, and *R*- factors based on ALL data will be even larger.

### Fractional atomic coordinates and isotropic or equivalent isotropic displacement parameters (Å^2^)

**Table:**

	*x*	*y*	*z*	*U*_iso_*/*U*_eq_	
S1	0.55009 (3)	0.30537 (10)	0.19986 (3)	0.0550 (3)	
O1	0.62688 (7)	−0.2687 (2)	0.09537 (7)	0.0517 (5)	
N1	0.47339 (13)	0.5797 (5)	0.05475 (13)	0.0976 (10)	
N2	0.55845 (11)	0.0864 (5)	0.04725 (11)	0.0593 (7)	
N3	0.61458 (10)	−0.0165 (4)	0.26076 (9)	0.0521 (6)	
N4	0.65779 (8)	−0.3092 (3)	0.19499 (8)	0.0432 (5)	
C1	0.50228 (12)	0.4550 (5)	0.08608 (13)	0.0643 (8)	
C2	0.53698 (10)	0.3009 (4)	0.12313 (11)	0.0501 (6)	
C3	0.56239 (10)	0.1264 (4)	0.10435 (11)	0.0456 (6)	
C4	0.59338 (10)	−0.0064 (3)	0.15318 (10)	0.0423 (6)	
C5	0.59027 (10)	0.0711 (3)	0.20748 (11)	0.0436 (6)	
C6	0.62674 (10)	−0.1949 (4)	0.15031 (10)	0.0422 (6)	
C7	0.66194 (10)	−0.4503 (4)	0.10807 (11)	0.0464 (6)	
C8	0.67552 (12)	−0.5875 (4)	0.06869 (12)	0.0610 (7)	
H8	0.6620	−0.5669	0.0277	0.073*	
C9	0.71069 (12)	−0.7582 (4)	0.09423 (14)	0.0657 (8)	
H9	0.7211	−0.8572	0.0697	0.079*	
C10	0.73088 (11)	−0.7869 (4)	0.15490 (13)	0.0607 (7)	
H10	0.7548	−0.9039	0.1701	0.073*	
C11	0.71652 (10)	−0.6462 (4)	0.19378 (12)	0.0539 (7)	
H11	0.7302	−0.6660	0.2348	0.065*	
C12	0.68079 (10)	−0.4743 (3)	0.16885 (10)	0.0433 (6)	
C13	0.61259 (11)	0.0713 (4)	0.31737 (10)	0.0462 (6)	
H13	0.5760	0.1493	0.3105	0.055*	
C14	0.61355 (13)	−0.1124 (4)	0.36007 (12)	0.0614 (7)	
H14A	0.5798	−0.2028	0.3439	0.074*	
H14B	0.6476	−0.2005	0.3642	0.074*	
C15	0.61409 (14)	−0.0298 (4)	0.42067 (12)	0.0698 (8)	
H15A	0.5779	0.0433	0.4173	0.084*	
H15B	0.6173	−0.1511	0.4475	0.084*	
C16	0.66333 (14)	0.1232 (4)	0.44618 (12)	0.0736 (9)	
H16A	0.6997	0.0473	0.4530	0.088*	
H16B	0.6614	0.1777	0.4841	0.088*	
C17	0.66063 (15)	0.3097 (4)	0.40402 (12)	0.0768 (9)	
H17A	0.6934	0.4046	0.4203	0.092*	
H17B	0.6256	0.3922	0.3997	0.092*	
C18	0.66133 (12)	0.2272 (4)	0.34334 (12)	0.0625 (8)	
H18A	0.6580	0.3485	0.3165	0.075*	
H18B	0.6979	0.1559	0.3473	0.075*	
H1N2	0.5767 (13)	−0.030 (4)	0.0372 (12)	0.076 (10)*	
H2N2	0.5435 (12)	0.177 (4)	0.0194 (13)	0.075 (10)*	
H1N3	0.6341 (11)	−0.132 (4)	0.2608 (11)	0.067 (9)*	

### Atomic displacement parameters (Å^2^)

**Table:**

	*U*^11^	*U*^22^	*U*^33^	*U*^12^	*U*^13^	*U*^23^
S1	0.0570 (5)	0.0570 (4)	0.0565 (5)	0.0136 (3)	0.0255 (3)	0.0145 (3)
O1	0.0574 (11)	0.0541 (10)	0.0389 (10)	0.0030 (8)	0.0075 (8)	0.0010 (8)
N1	0.106 (2)	0.111 (2)	0.084 (2)	0.0561 (18)	0.0410 (17)	0.0446 (17)
N2	0.0645 (16)	0.0656 (17)	0.0407 (16)	0.0077 (13)	0.0052 (12)	0.0114 (13)
N3	0.0647 (15)	0.0519 (14)	0.0394 (14)	0.0158 (11)	0.0155 (11)	0.0076 (11)
N4	0.0461 (12)	0.0434 (11)	0.0366 (12)	0.0030 (9)	0.0070 (9)	0.0029 (9)
C1	0.0602 (19)	0.0753 (19)	0.064 (2)	0.0181 (15)	0.0288 (15)	0.0232 (16)
C2	0.0437 (15)	0.0552 (15)	0.0529 (17)	0.0115 (12)	0.0168 (12)	0.0214 (13)
C3	0.0393 (14)	0.0542 (15)	0.0410 (16)	−0.0047 (11)	0.0085 (12)	0.0104 (12)
C4	0.0433 (14)	0.0424 (13)	0.0383 (15)	−0.0018 (11)	0.0078 (11)	0.0054 (11)
C5	0.0418 (14)	0.0443 (13)	0.0446 (16)	−0.0004 (11)	0.0126 (12)	0.0072 (12)
C6	0.0422 (14)	0.0460 (13)	0.0370 (15)	−0.0083 (11)	0.0096 (11)	−0.0027 (12)
C7	0.0466 (15)	0.0455 (14)	0.0460 (16)	−0.0011 (11)	0.0122 (12)	−0.0024 (12)
C8	0.0661 (19)	0.0665 (17)	0.0491 (17)	−0.0009 (15)	0.0154 (14)	−0.0073 (14)
C9	0.0667 (19)	0.0626 (18)	0.072 (2)	0.0009 (15)	0.0280 (17)	−0.0172 (15)
C10	0.0555 (17)	0.0545 (16)	0.074 (2)	0.0063 (13)	0.0223 (15)	0.0004 (15)
C11	0.0540 (17)	0.0553 (16)	0.0504 (17)	0.0060 (13)	0.0124 (13)	0.0057 (13)
C12	0.0424 (15)	0.0441 (14)	0.0423 (16)	−0.0066 (11)	0.0111 (12)	−0.0022 (11)
C13	0.0537 (16)	0.0468 (14)	0.0431 (16)	0.0072 (12)	0.0221 (12)	0.0067 (11)
C14	0.085 (2)	0.0515 (15)	0.0513 (18)	−0.0059 (14)	0.0261 (15)	0.0081 (13)
C15	0.103 (2)	0.0629 (17)	0.0560 (19)	0.0047 (17)	0.0423 (17)	0.0149 (14)
C16	0.105 (3)	0.0748 (19)	0.0445 (18)	0.0059 (18)	0.0274 (17)	−0.0021 (15)
C17	0.109 (3)	0.0680 (19)	0.059 (2)	−0.0191 (17)	0.0346 (18)	−0.0087 (16)
C18	0.078 (2)	0.0593 (16)	0.0576 (18)	−0.0065 (14)	0.0325 (15)	0.0038 (14)

### Geometric parameters (Å, º)

**Table:**

S1—C5	1.727 (3)	C9—H9	0.9300
S1—C2	1.740 (3)	C10—C11	1.383 (3)
O1—C6	1.375 (3)	C10—H10	0.9300
O1—C7	1.387 (3)	C11—C12	1.387 (3)
N1—C1	1.150 (3)	C11—H11	0.9300
N2—C3	1.343 (3)	C13—C18	1.506 (3)
N2—H1N2	0.92 (3)	C13—C14	1.515 (3)
N2—H2N2	0.86 (3)	C13—H13	0.9800
N3—C5	1.332 (3)	C14—C15	1.514 (4)
N3—C13	1.455 (3)	C14—H14A	0.9700
N3—H1N3	0.86 (2)	C14—H14B	0.9700
N4—C6	1.304 (3)	C15—C16	1.502 (4)
N4—C12	1.394 (3)	C15—H15A	0.9700
C1—C2	1.392 (4)	C15—H15B	0.9700
C2—C3	1.382 (3)	C16—C17	1.513 (4)
C3—C4	1.430 (3)	C16—H16A	0.9700
C4—C5	1.391 (3)	C16—H16B	0.9700
C4—C6	1.434 (3)	C17—C18	1.524 (3)
C7—C8	1.370 (3)	C17—H17A	0.9700
C7—C12	1.375 (3)	C17—H17B	0.9700
C8—C9	1.378 (4)	C18—H18A	0.9700
C8—H8	0.9300	C18—H18B	0.9700
C9—C10	1.377 (4)		
			
C5—S1—C2	90.89 (11)	C7—C12—C11	119.6 (2)
C6—O1—C7	104.00 (17)	C7—C12—N4	109.3 (2)
C3—N2—H1N2	120.8 (18)	C11—C12—N4	131.2 (2)
C3—N2—H2N2	122.0 (19)	N3—C13—C18	111.91 (19)
H1N2—N2—H2N2	116 (3)	N3—C13—C14	109.3 (2)
C5—N3—C13	125.8 (2)	C18—C13—C14	111.0 (2)
C5—N3—H1N3	115.3 (18)	N3—C13—H13	108.2
C13—N3—H1N3	118.9 (18)	C18—C13—H13	108.2
C6—N4—C12	104.58 (19)	C14—C13—H13	108.2
N1—C1—C2	178.9 (4)	C15—C14—C13	111.6 (2)
C3—C2—C1	125.4 (2)	C15—C14—H14A	109.3
C3—C2—S1	112.71 (17)	C13—C14—H14A	109.3
C1—C2—S1	121.8 (2)	C15—C14—H14B	109.3
N2—C3—C2	124.1 (2)	C13—C14—H14B	109.3
N2—C3—C4	124.2 (2)	H14A—C14—H14B	108.0
C2—C3—C4	111.7 (2)	C16—C15—C14	111.8 (2)
C5—C4—C3	112.4 (2)	C16—C15—H15A	109.3
C5—C4—C6	120.9 (2)	C14—C15—H15A	109.3
C3—C4—C6	126.7 (2)	C16—C15—H15B	109.3
N3—C5—C4	126.6 (2)	C14—C15—H15B	109.3
N3—C5—S1	121.08 (19)	H15A—C15—H15B	107.9
C4—C5—S1	112.33 (17)	C15—C16—C17	110.3 (3)
N4—C6—O1	114.6 (2)	C15—C16—H16A	109.6
N4—C6—C4	126.9 (2)	C17—C16—H16A	109.6
O1—C6—C4	118.5 (2)	C15—C16—H16B	109.6
C8—C7—C12	124.6 (2)	C17—C16—H16B	109.6
C8—C7—O1	127.8 (2)	H16A—C16—H16B	108.1
C12—C7—O1	107.6 (2)	C16—C17—C18	110.5 (2)
C7—C8—C9	115.1 (3)	C16—C17—H17A	109.6
C7—C8—H8	122.5	C18—C17—H17A	109.6
C9—C8—H8	122.5	C16—C17—H17B	109.6
C10—C9—C8	122.2 (3)	C18—C17—H17B	109.6
C10—C9—H9	118.9	H17A—C17—H17B	108.1
C8—C9—H9	118.9	C13—C18—C17	111.5 (2)
C9—C10—C11	121.7 (3)	C13—C18—H18A	109.3
C9—C10—H10	119.2	C17—C18—H18A	109.3
C11—C10—H10	119.2	C13—C18—H18B	109.3
C10—C11—C12	116.9 (2)	C17—C18—H18B	109.3
C10—C11—H11	121.5	H18A—C18—H18B	108.0
C12—C11—H11	121.5		
			
N1—C1—C2—C3	25 (16)	C3—C4—C6—O1	−4.7 (3)
N1—C1—C2—S1	−152 (16)	C6—O1—C7—C8	178.5 (2)
C5—S1—C2—C3	0.11 (19)	C6—O1—C7—C12	−0.3 (2)
C5—S1—C2—C1	177.5 (2)	C12—C7—C8—C9	−0.1 (4)
C1—C2—C3—N2	2.4 (4)	O1—C7—C8—C9	−178.7 (2)
S1—C2—C3—N2	179.76 (19)	C7—C8—C9—C10	−0.5 (4)
C1—C2—C3—C4	−177.6 (2)	C8—C9—C10—C11	0.5 (4)
S1—C2—C3—C4	−0.3 (3)	C9—C10—C11—C12	0.0 (4)
N2—C3—C4—C5	−179.7 (2)	C8—C7—C12—C11	0.6 (4)
C2—C3—C4—C5	0.3 (3)	O1—C7—C12—C11	179.46 (19)
N2—C3—C4—C6	2.7 (4)	C8—C7—C12—N4	−178.7 (2)
C2—C3—C4—C6	−177.3 (2)	O1—C7—C12—N4	0.2 (3)
C13—N3—C5—C4	177.6 (2)	C10—C11—C12—C7	−0.5 (3)
C13—N3—C5—S1	−2.1 (3)	C10—C11—C12—N4	178.6 (2)
C3—C4—C5—N3	−180.0 (2)	C6—N4—C12—C7	0.0 (2)
C6—C4—C5—N3	−2.2 (4)	C6—N4—C12—C11	−179.1 (2)
C3—C4—C5—S1	−0.2 (3)	C5—N3—C13—C18	−88.0 (3)
C6—C4—C5—S1	177.51 (16)	C5—N3—C13—C14	148.6 (2)
C2—S1—C5—N3	179.8 (2)	N3—C13—C14—C15	177.6 (2)
C2—S1—C5—C4	0.06 (19)	C18—C13—C14—C15	53.7 (3)
C12—N4—C6—O1	−0.3 (2)	C13—C14—C15—C16	−55.2 (3)
C12—N4—C6—C4	179.4 (2)	C14—C15—C16—C17	56.8 (3)
C7—O1—C6—N4	0.4 (2)	C15—C16—C17—C18	−57.1 (3)
C7—O1—C6—C4	−179.33 (19)	N3—C13—C18—C17	−177.2 (2)
C5—C4—C6—N4	−1.7 (4)	C14—C13—C18—C17	−54.8 (3)
C3—C4—C6—N4	175.7 (2)	C16—C17—C18—C13	56.8 (3)
C5—C4—C6—O1	178.0 (2)		

### Hydrogen-bond geometry (Å, º)

**Table:**

*D*—H···*A*	*D*—H	H···*A*	*D*···*A*	*D*—H···*A*
N2—H1*N*2···O1	0.92 (3)	2.13 (3)	2.783 (4)	127 (2)
N2—H2*N*2···N1^i^	0.86 (3)	2.25 (3)	3.088 (4)	166 (3)
N3—H1*N*3···N4	0.86 (2)	2.12 (3)	2.787 (3)	135 (2)

Symmetry code: (i) −*x*+1, −*y*+1, −*z*.
